# Bilateral Renal Mass-Renal Disorder: Tuberculosis

**DOI:** 10.1155/2013/724693

**Published:** 2013-09-15

**Authors:** Ozlem Tiryaki, Celalettin Usalan, Samet Alkan

**Affiliations:** ^1^Department of Nephrology, Gaziantep University School of Medicine, Gaziantep, Turkey; ^2^Department of Internal Medicine, Gaziantep University School of Medicine, Gaziantep, Turkey

## Abstract

A 30-year-old woman has presented complaining of weakness and fatigue to her primary care physician. The renal sonography is a routine step in the evaluation of new onset renal failure. When the renal masses have been discovered by sonography in this setting, the functional imaging may be critical. We reported a case about bilateral renal masses in a young female patient with tuberculosis and renal insufficiency. Magnetic resonance (MR) has revealed the bilateral renal masses in patient, and this patient has been referred to our hospital for further management. The patient's past medical and surgical history was unremarkable.

## 1. Introduction

The end-stage renal disease (ESRD) is a well-documented risk factor for developing an infection with *Mycobacterium tuberculosis* [1]. The presentation of *Mycobacterium tuberculosis* in patients with end-stage renal disease depends on the degree of immunosuppression that it could be atypical and difficult to diagnose compared with the classical presentation of *Mycobacterium tuberculosis* in nonimmunocompromised individuals.

## 2. Case Report

A 30-year-old female patient was found to have a creatinine of 4.8 mg/dL on routine preemployment health checkup. She had no facial puffiness, swelling of legs, hematuria, or dysuria, and she denied any history of fever, joint pains, weight loss, or consumption of indigenous medicines. There was neither a regular medication history nor a particular characteristic in the family history. Physical examination was normal except for mild pallor and tachycardia. Her physical examination revealed that her overall condition was in between, and she was conscious and cooperating. Her blood pressure was 110/70 mm/Hg, pulse rate 106/min, and fever 36.2°C. Other system examinations were all normal. The initial laboratory studies revealed a hypochromic microcyter anemia. There was no atypical cell on peripheral blood smear. Erythrocyte sedimentation rate (ESR) was 40 mm/h. PTH 279 was pg/mL. No pathologic findings were observed in urinalysis. The patient was hospitalized in nephrology service with a diagnosis of bilateral renal mass. PPD was positive (15 mm diameter). No fever was recorded during the follow-up period. Patient's laboratory data are depicted in the Table 1. 

**Figure 1:**
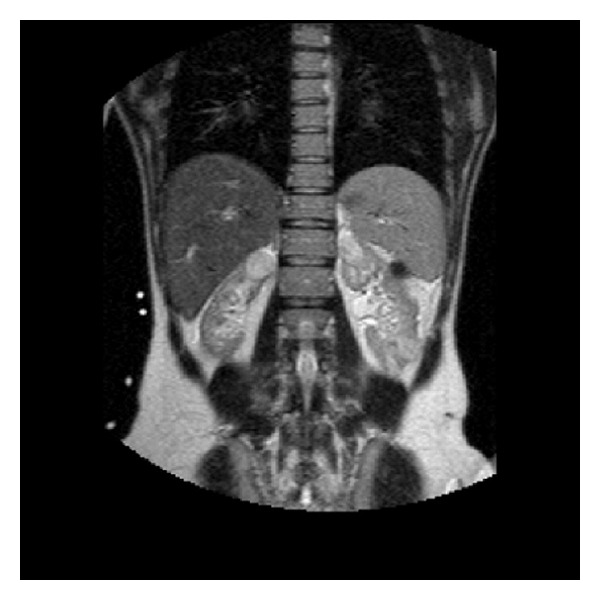
T2-W coronal MR image demonstrates a large right renal mass and numerous left renal masses, some of which were not evident on the US.

The renal sonography has demonstrated small kidneys according to her age and bilateral renal masses. The hyperechoic right renal mass is measured 36∗30 mm, and hyperechoic left renal mass is measured 42∗40 mm. The contrast MR examination was performed on the same day. These renal masses were slightly hyperintense in renal cortex on both T1- and T2-weighed images. There was remarkable thinning of the right renal cortex and the left renal cortex with multiple masses (Figure 1).

**Figure 2:**
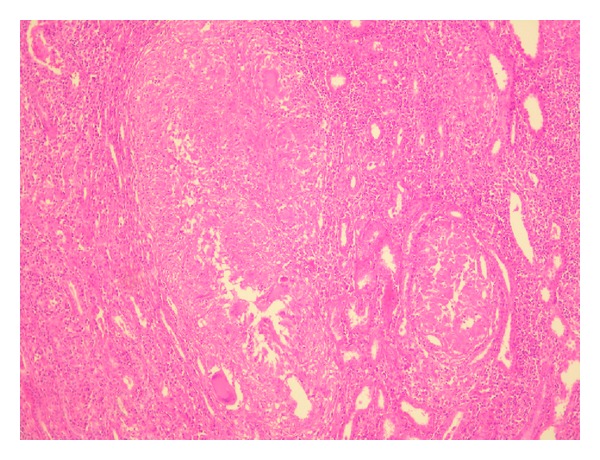
Kidney biopsy on light microscopy. Caseating epithelioid cells with granulomas and Langhans giant cells on renal tuberculosis. Hematoxylin-eosin stain, original magnification ×100.

We have diagnosed this patient by ultrasound-guided percutaneous biopsy on the upper pole of the left kidney mass. When we examined the biopsy specimen on light microscopy (Figure 2), we have seen marked infiltration by lymphocytes, scattered medium-sized caseating epithelioid cells with granulomas, and Langhans giant cells. Also, there was caseous necrosis in one of the granulomas.

Work up for caseating granulomas in interstitial nephritis has revealed normal serum angiotensin-converting enzyme levels and normal serum calcium levels. Serum polymerase chain reaction (PCR) for TB was positive.

After ruling out all other causes of caseating granulomatous nephritis, positive PPD and PCR test for TB were given with tuberculosis prevalence in this part of the Turkey. The provisional diagnosis of renal tuberculosis was made. The patient was started on antitubercular therapy with an initial 2-month intensive phase treatment which includes isoniazid (5 mg/kg/day), rifampin (10 mg/kg/day), ethambutol (20 mg/kg/day), and pyrazinamide (25 mg/kg/day) and followed by a 4-month continuation phase therapy with dosages adjusted to creatinine clearance. After starting the treatment, her serum creatinine level started to improve and settled at 2.6 mg/dL.

## 3. Discussion

There is an increased incidence of TB in ESRD compared to the general population. This is especially important in areas where the tuberculosis is endemic. The presentation of TB in uremic patients is relatively uncommon and insidious. Moreover, diagnosis and management of treatment have many special challenges for the physicians who are carrying out the treatment [2]. Extrapulmonary tuberculosis is common in patients with ESRD, and involvement of lymph nodes is the most common extrapulmonary presentation. Of the 296 patients undergoing hemodialysis regularly between 1980 and 1996, eighteen tuberculosis patients (6.08%) were reported by Taskapan et al. They have found extrapulmonary involvement in 7 patients (38%) their TB patients. The distribution of extrapulmonary TB in these seven patients was like that four patients (22.2%) with tuberculous lymphadenitis, two patients (11.1%) with tuberculous peritonitis, and one patient (5.5%) with urinary tuberculosis [3]. Diagnosis of tuberculous lymphadenitis is particularly difficult. 

The sensitivity of conventional methods used in mycobacteriology laboratories, for example, acid-fast bacillus smear examination and culture, may not exceed 40% [4, 5]. Culture obtained by means of a polymerase chain reaction (PCR) has the advantage of yielding a quick and sensitive result.

The differentiating feature of this case was its manifestation with bilateral renal mass, and no such case was found in the literature.

In this case, we have demonstrated that the patient presented a mass on the kidney without active pulmonary tuberculosis. If the clinical presentation is suspicious, the invasive procedures with tissue biopsy may be necessary. Follow up of extrapulmonary tuberculosis is very important in patients with end-stage renal disease.

## Figures and Tables

**Table 1:** Laboratory studies*.

Laboratory study	Result
CBC	
Hemoglobin, g/dL	8.5
Total leukocyte count, × 10^3^/L	5.6
Platelet count, × 10^3^/L	121
ESR, mm/h	40
CRP mg/L	9.6
Blood urea nitrogen, mg/dL	80
Serum creatinine, mg/dL	4.8
AST U/L	8
ALT U/L	6
Urinalysis	
pH	7.5
Protein	Negative
Glucose	Negative
Erythrocyte	Trace
Leucocyte	Negative
Leucocyte esterase	Negative
24 hours urine protein mg/day	260
Urine culture	Negative
Urine for AFB staining	Negative
Urine for AFB culture	Negative
Plain radiography	Unremarkable
Chest radiography	Unremarkable
Anti-HIV antibody	Negative
HBs antigen	Negative
Anti-HCV antibody	Negative
ANA	Negative
Double-stranded DNA	Negative
pANCA	Negative
cANCA	Negative
Complement 3	Normal
Complement 4	Normal

*AFB indicates acid-fast bacilli; HIV: human immunodeficiency virus; HBs: hepatitis B surface; HCV: hepatitis C virus; ANA: antinuclear antibody; pANCA: perinuclear antineutrophil cytoplasmic antibody; and cANCA: cytoplasmic antineutrophil cytoplasmic antibody.
